# Characteristics of Long‐Term Femoral Neck Bone Loss in Postmenopausal Women: A 25‐Year Follow‐Up

**DOI:** 10.1002/jbmr.4444

**Published:** 2021-10-19

**Authors:** Anna Moilanen, Juho Kopra, Heikki Kröger, Reijo Sund, Toni Rikkonen, Joonas Sirola

**Affiliations:** ^1^ Kuopio Musculoskeletal Research Unit (KMRU), Institute of Clinical Medicine, School of Medicine University of Eastern Finland (UEF) Kuopio Finland; ^2^ Department of Orthopaedics, Traumatology and Hand Surgery Kuopio University Hospital Kuopio Finland

**Keywords:** OSTEOPOROSIS, BONE LOSS, MENOPAUSE, DXA, HORMONE REPLACEMENT, CORTICOSTEROIDS

## Abstract

The aim of this study was to monitor long‐term changes in bone mineral density (BMD) after menopause and factors affecting BMD. The study population consisted of a random sample of 3222 women from the Kuopio Osteoporosis Risk Factor and Prevention (OSTPRE) study, of which 62.1% were postmenopausal at the beginning of the study. This group of women underwent dual‐energy X‐ray absorptiometry (DXA) measurements at the femoral neck every 5 years from baseline (in 1989) up to 25‐year follow‐up. They also responded to risk‐factor questionnaires at 5‐year intervals. During the 25‐year follow‐up, the baseline cohort decreased to 686 women. The women were divided into quartiles based on their baseline BMD. Self‐reported hormone replacement therapy (HRT) and corticosteroid use were divided into ever users and never users. Morbidity was assessed as the total number of self‐reported diseases and BMD‐affecting diseases. The mean 25‐year BMD change was found to be −10.1%, *p* < 0.001. Higher baseline BMD was associated with higher bone loss rate; the reduction in the highest quartile BMD was 11.1% and in the lowest quartile 7.4% (*p* = 0.0031). Lower baseline body mass index (BMI) and a greater increase in BMI were found to protect against postmenopausal bone loss (*p* < 0.001). The lowest bone loss quartile included 15.2% more HRT users than the highest bone loss quartile (*p* = 0.004). The number of diseases/bone‐affecting diseases, use of vitamin D/calcium supplementation, use of corticosteroids, smoking or alcohol use had no statistical significance for annual bone loss rate. This study presents hitherto the longest (25‐year) BMD follow‐up in postmenopausal women. The linear femoral neck bone loss of 10% was less than previously assumed. A 5‐year BMD change appeared to predict long‐term bone loss in postmenopausal women. © 2021 The Authors. *Journal of Bone and Mineral Research* published by Wiley Periodicals LLC on behalf of American Society for Bone and Mineral Research (ASBMR).

## Introduction

Osteoporosis is the most common metabolic bone disease^(^ ^1^ ^)^ and is characterized by low bone mineral density (BMD). It appears more commonly among women than men, due to the changes occurring after menopause and to women's lower peak bone mass.^(^ ^2^ ^)^ According to previous studies, women with osteoporosis have more comorbidities and medications than women without osteoporosis.^(^ ^3^ ^)^ More precisely, numerous risk factors are related to decreased BMD and osteoporosis, for example, certain lifestyle factors, age, low body mass index (BMI), and long‐term use of bone‐affecting medication.^(^ ^4^ ^)^ Postmenopausal status also increases the risk of osteoporotic BMD; bone loss accelerates considerably especially at the onset of menopause.^(^ ^5^, ^6^ ^)^

Previous studies have shown that bone loss increases at the time of menopausal transition^(^ ^7^ ^)^ and decreases in later life.^(^ ^5^, ^6^, ^8^ ^)^ It has also been reported that the decline in BMD at the femoral neck is almost linear with age over a 15‐year period for postmenopausal women.^(^ ^9^ ^)^ The use of hormone replacement therapy (HRT) appears to decrease bone loss.^(^ ^9^, ^10^, ^11^ ^)^ However, knowledge of very long‐term changes (over 15 years) in BMD at the population level has not previously been available. The ability of short‐term bone loss to predict long‐term bone loss at the population level is unknown.

The golden standard for measuring bone mass is central dual‐energy X‐ray absorptiometry (DXA). A DXA measurement of the lumbar spine is possibly prone to falsifying factors, such as degenerative changes or vertebral compression fractures, which may affect the BMD value.^(^ ^12^ ^)^ Therefore, femoral neck BMD is considered more reliable in an elderly population and is primarily used for the diagnosis of osteoporosis.

The aim of this study was to examine the characteristics of 25‐year changes in femoral BMD after menopause. We investigated associations between the rate of bone loss and selected risk factors (BMI and BMI change, number of diseases, bone‐affecting diseases, use of HRT and corticosteroids, use of vitamin D and calcium supplementation, alcohol use, smoking, age, rheumatoid arthritis, and postmenopausal status at baseline).

## Subjects and Methods

This study is based on the ongoing Kuopio Osteoporosis Risk Factor and Prevention (OSTPRE) study (https://sites.uef.fi/kmru/ostpre/). The OSTPRE study investigated the significance of lifestyle and health disorders for BMD and the susceptibility of perimenopausal to postmenopausal women to falls and fractures.^(^ ^5^ ^)^ The study group consisted of all women living in the Kuopio region, Eastern Finland, and born between 1932 and 1941 (*n* = 14,220).^(^ ^13^, ^14^ ^)^ The baseline postal inquiry was sent in 1989, and enquired about health status, physical activity, nutrition, alcohol consumption, smoking habits, medication, use of HRT and fracture history. The study was carried out by sending follow‐up inquiries at 5‐year intervals in 1994, 1999, 2004, 2009, and 2014. In addition to inquiries, a randomly selected population‐based sample of women (*n* = 3222) was invited to undergo DXA at 5‐year intervals at Kuopio University Hospital and the University of Eastern Finland's Kuopio campus. At baseline (in 1989), valid DXA measurement results were obtained for 2695 women. Some of the women were not willing to participate (12.3%), and some had factors inducing DXA measurement errors (eg, hip implants, spine degeneration, morbid obesity) that would have made the measurement unreliable (4.1%). The follow‐up measurements were carried out after 5 (*n* = 2583), 10 (*n* = 2482), 15 (*n* = 2135), 20 (*n* = 1305), and 25 years (*n* = 686). The causes for dropout were unwillingness to participate, erroneous DXA measurement, healthcare institutionalization, or death. The mortality and need for long‐term care (ie, healthcare institutionalization) of the OSTPRE population during the follow‐up are described in Supplemental Table S1 in greater detail. The final study population consisted of women with valid DXA measurements from the femoral neck during the 25‐year follow‐up. The study was approved by the ethics committee of the University of Eastern Finland and Kuopio University hospital.

During the 25‐year follow‐up, four DXA instruments (Lunar, Madison, WI, USA) were used: DPX, DPX‐IQ, Prodigy, and iDXA. A Lunar DPX scanner was used at baseline and at the 5‐year measurement, DPX‐IQ at the 10‐year measurement, Prodigy (GE Medical Systems, Lunar, Liege Belgium) at the 15‐year measurement, and iDXA thereafter (GE Medical Systems, Lunar, Liege, Belgium).

The DPX scanner was replaced with a DPX‐IQ instrument during the 10‐year measurement. For 30% of the participants, the 10‐year DXA measurement was performed with the DPX‐IQ instead of the DPX. A total of 90 women were scanned with both instruments on the same day. In both hip and spine DXA, a high linear correlation (*r* spine = 0.990, *n* = 88; *r* neck = 0.974, *n* = 90) was found for the BMD values between the scanners. Because direct cross‐calibration between the DPX and the Prodigy was not possible owing to the unavailability of the original instrument, similar correlations were calculated in order to reveal differences between DPX‐IQ and Prodigy DXA. Combining the cross‐calibration between the DPX‐IQ and Prodigy from two scans of the lumbar spine L_1_–L_4_ (*n* = 29) and proximal femur (*n* = 28) with previous DPX and DPX‐IQ cross‐calibration data, the final calibration between the DPX and Prodigy could be conducted. This was possible because the relationships between BMD values of DPX‐IQ and Prodigy scanners demonstrated high linearity and *r* values (*r* neck = 0.993, *r* Ward's = 0.988, *r* trochanter = 0.991, *r* total = 0.996). The *r* values are comparable with those between DPX and DPX‐IQ scanners; this high linear correlation in BMD has been presented in detail.^(^ ^15^ ^)^ Final corrections between the DPX and Prodigy were calculated by mathematically combining DPX versus DPX‐IQ and DPX‐IQ versus Prodigy correction functions. The use of patient measurements rather than phantom measurements for calibration was emphasized.

### Lunar Prodigy versus iDXA

In order to reveal the association and agreement between the measurements of 15‐year DXA (Prodigy) and 20‐year (iDXA) densitometers, the data were analyzed by using linear regression analysis, Deming regression, paired *t* test, Pearson's correlation analysis, and Bland and Altman analysis.^(^ ^16^ ^)^ For scatter plots the statistical significance of the intercept of each regression line was tested. If the intercept was not different from zero, the regression analysis was repeated with the intercept forced through the origin.^(^ ^17^ ^)^ The accuracy of corrections, obtained with a regression line, was expressed as the standard error of the estimates. The Bland and Altman method was used to evaluate the bias in results between the devices.^(^ ^16^ ^)^ Hologic anthropometric lumbar spine phantom, European Spine Phantom (ESP), and GE Healthcare Lunar aluminum spine phantom^(^ ^18^, ^19^ ^)^ were scanned 10 times during a period of 1 week to calculate the short‐term precision error (coefficients of variation, CV% = (standard deviation [SD]/mean) × 100%) of the instruments.^(^ ^20^ ^)^

These calculations have been described.^(^ ^21^, ^22^, ^23^, ^24^ ^)^ All values were converted to match DPX‐IQ in the present analyses.

The use of HRT, including estrogen‐containing tablets or plasters, and the use of corticosteroids (yes/no), was categorized into ever use (regular or occasional) and never use during the 25‐year follow‐up, based on self‐reports in the follow‐up postal enquiries. In addition, the duration of HRT was enquired based on self‐reports. The total numbers of self‐reported diseases and self‐reported bone‐affecting diseases were calculated. The bone‐affecting diseases included diabetes, chronic renal and liver diseases, asthma, lactose malabsorption, celiac disease, endocrine abnormalities (thyroid/parathyroid glands, adrenals), alcoholism, reactive arthritis, multiple sclerosis, inflammatory bowel diseases, lymphoma, leukemia, connective tissue disease, skeletal developmental disorder, vitamin deficiency rickets, long‐term depression, chronic obstructive pulmonary disease (COPD), osteomalacia, bone cancer, and myelodysplastic syndromes (MDS).^(^ ^25^ ^)^ Weight and height were measured at every follow‐up using a calibrated scale at the time of DXA measurements. BMI was calculated as weight (kg)/height squared (m^2^). A change in BMI was calculated as the difference between baseline and the 25‐year measurement.

The use of dairy products was inquired with questions about the daily use of milk and cheese products. In addition, the use of calcium and vitamin D supplementation (yes/no) was inquired separately apart from dairy intake of calcium. The use of alcohol was calculated based on self‐reports of alcohol products on daily basis (dL/day). Smoking was inquired based on the question about current use of tobacco, pipe, or cigars (yes/no). In addition, the participant history of any low‐trauma energy fracture or hip fractures of the parents (yes/no) was inquired with specific questions.

### Statistical methods

The explorative analysis of BMD was carried out by analyzing the data with SPSS version 25 for Windows (IBM Corp., Armonk, NY, USA). A linear estimate based on repeated BMD measurements was used to obtain the age‐dependent equation for BMD. The annual bone loss rate was calculated as a percentage of baseline BMD and with the formula [(BMD at the follow‐up year – BMD at baseline)/duration of follow‐up]. The study population was divided into bone loss quartiles (FQ1–FQ4); FQ1 had the highest bone loss rate and FQ4 had the lowest rate. Analyses of contingency tables were conducted using the chi‐square test without Yates' correction in order to compare the distribution of risk factors between bone loss quartiles. The effect of continuous risk factors on the 25‐year bone loss rate was investigated with analysis of variance (ANOVA).

The femoral neck *T*‐score value was determined according to the Third National Health and Nutrition Examination Survey (NHANES III) with reference to white women aged 20 to 29 years.^(^ ^26^ ^)^

## Results

Table 1 describes the characteristics of the study population at baseline and in the 25‐year follow‐up. The mean age of the study population at baseline was 53.3 years (range, 47–59 years), and 62.1% (*n* = 2002) of the women were already postmenopausal. The duration of the follow‐up ranged from 23.6 to 25.9 years. Statistically significant differences were observed in BMD, age, height, weight, dairy product use, smoking, previous fracture, any use of alcohol, rheumatoid arthritis, osteoporosis, calcium or vitamin D supplement, corticosteroid use, and bone‐affecting diseases. The cumulative mortality and long‐term care (healthcare institutionalization) during the 25‐year follow‐up of the total OSTPRE population are described in Supplemental Table S1. The cumulative mortality of the OSTPRE population was 16.6% and the need for long‐term care (healthcare institutionalization) 9.5%, adding up to an overall 26.1% dropout percentage. Accordingly, the total number of dropouts due to these reasons was 2695*0.261 = 703. The total number of the full measurement sample dropouts was 2695–686 = 2009. This adds up to the explained dropout rate of 35% (703/2009) of the measurement sample for these reasons.

**Table 1:** Characteristics of the Study Population at Baseline and at 25‐Year Follow‐Up

Continuous variables	Baseline (*n* = 2695)	25‐Year follow‐up (*n* = 686)	*p* *
BMD (g/cm^2^), mean ± SD	0.93 ± 0.13	0.85 ± 0.13	<0.001
Age (years), mean ± SD	53.3 ± 2.8	77.4 ± 2.8	<0.001
Height (cm), mean ± SD	161.2 ± 5.2	157.8 ± 5.2	<0.001
Weight (kg), mean ± SD	68.4 ± 11.5	70.0 ± 13.0	0.002
Dairy products use (dL/d), mean ± SD	4.2 ± 2.7	9.2 ± 70.5	<0.001
Categorical variables, %			
Previous fracture	21.5	37.3	<0.001
Smoking	11.7	2.5	<0.001
Any use of alcohol (%)	37.7	44.2	0.002
Rheumatoid arthritis	4.6	2.5	0.013
Osteoporosis	1.6	12.2	<0.001
Calcium or D‐vitamin supplement	10.3	78.7	<0.001
Corticosteroid use	4.3	7.6	<0.001
Diseases affecting bones	26.6	45.0	<0.001
Hip fracture in parents	Not available	12.0	

ANOVA = analysis of variance.

* Statistical significance obtained with ANOVA for continuous variables, chi‐square for categorical variables.

### BMD during 25 years

**Fig 1:**
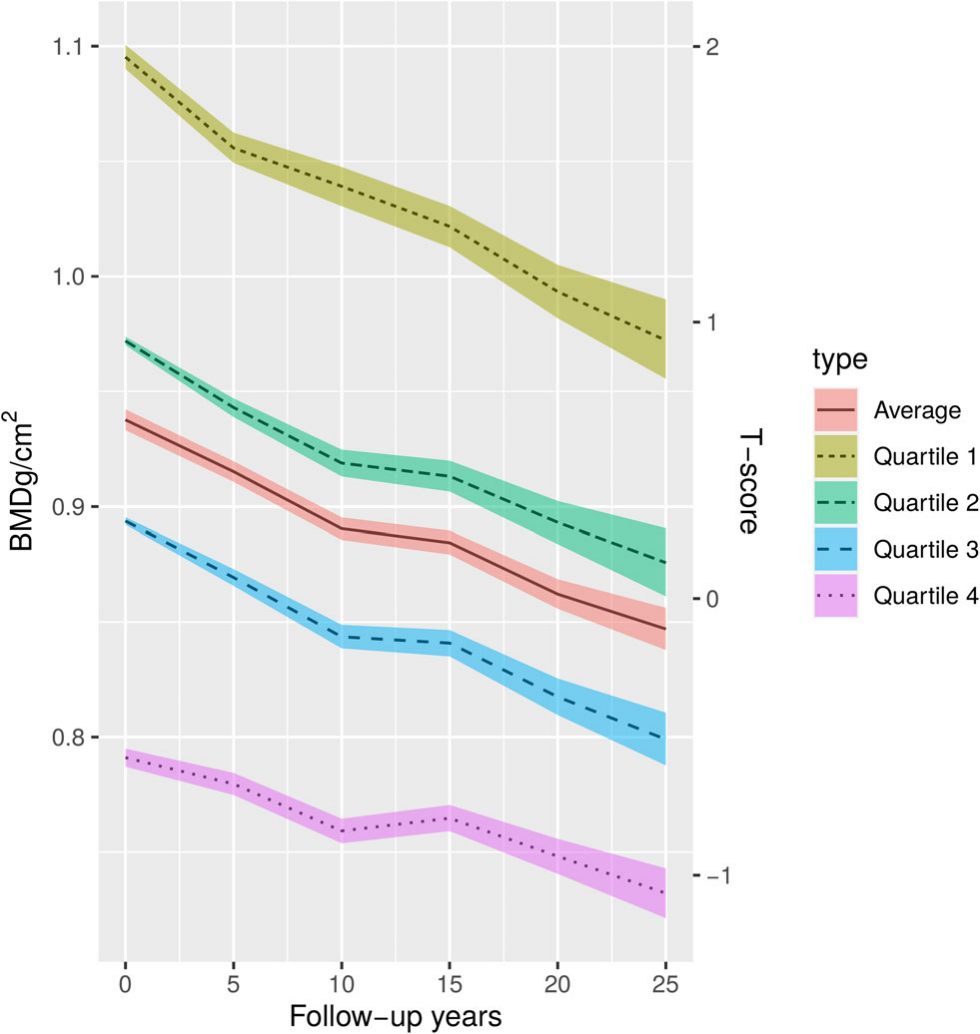
Trends in absolute BMD (95% CI) measured at the femoral neck during the 25‐year follow‐up with respect to baseline BMD quartiles. The number of women at each follow‐up: baseline (*n* = 2695), 5 years (*n* = 2583), 10 years (*n* = 2482), 15 years (*n* = 2135), 20 years (*n* = 1305), and 25 years (*n* = 686).

Figure 1 describes the observed femoral neck BMD of the study cohort from baseline (*n* = 2695) up to the 25‐year measurement (*n* = 686). The total observed change in BMD was −10.1% at constant rate. The observed 25‐year bone loss of the population with no self‐reported diseases or medications (*n* = 105 at 25‐year follow‐up [FU]) was 9.7% (data not shown). Linearity was tested using a simple linear regression for absolute BMD with a second‐order polynomial of time from baseline and also using age as a covariate. The second‐order polynomial was found to be statistically insignificant (beta = 0.07247, *t* = 0.507, *p* = 0.612), indicating linearity. In order to reveal possible long‐term trends in BMD change, the BMD change from baseline was monitored and found to be −4.7% up to 10 years and −6.0% between 15 and 25 years.

The study population was also divided into baseline BMD quartiles (BMD‐Q1 to BMD‐Q4, Fig. 1). A higher baseline BMD was associated with a higher 25‐year bone loss (difference between 25‐year and baseline BMD: BMD‐Q1 = 0.12 g/cm^2^ versus BMD‐Q4 = 0.059 g/cm^2^). However, within the highest baseline BMD quartile (BMD‐Q1), women's BMD did not decrease to the baseline mean of the total population (0.973 versus 0.938 g/cm^2^). Figure 1 also describes the *T*‐scores of the quartiles according to NHANES III criteria.

On average the linear relationship between BMD and age was BMD = 0.9386209–0.0040006*(age‐53.3), where the value 0.9386209 is the average BMD of 53.3‐year‐old women; 53.3 years represents the average age of patients at the time of the baseline measurement.

### Risk factors for bone loss

The mean annual bone loss rate of the study population was 0.4%. The association of selected risk factors with 25‐year bone loss is described in Table 2. The study population was divided into follow‐up quartiles (FQ1–FQ4) based on 25‐year bone loss and reported as a percentage of baseline BMD: FQ1: 0.9%, FQ2: 0.5%, FQ3: 0.3%, and FQ4: 0.1%. Lower baseline BMI and an increase in BMI during the follow‐up were associated with a lower 25‐year bone loss rate (*p* < 0.001). However, initial baseline age was not significantly associated with bone loss rate.

**Table 2:** Factors Affecting Bone Loss During the 25‐Year Follow‐Up According to 25‐Year Annual Bone Loss Rate Quartiles

Factor	FQ1 (*n* = 171)	FQ2 (*n* = 172)	FQ3 (*n* = 172)	FQ4 (*n* = 171)	All (*n* = 686)	Significance
Age, baseline (years)	52.9	52.5	52.5	52.7	53.4	*p* = 0.55
Baseline BMI (kg/m^2^)	26.7	25.9	25.5	24.9	26.5	*p* < 0.001
Postmenopausal at baseline (%)	58.2	52.0	60.9	58.0	62.1	*p* = 0.63
Any use of alcohol at baseline (%)	36.3	41.9	45.9	46.2	42.6	*p* = 0.21
Smoking, at baseline (%)	23.4	18.0	20.9	21.6	21.0	*p* = 0.67
Calcium or vitamin D supplement (%)	7.0	9.3	12.2	13.5	10.5	*p* = 0.20
Rheumatoid arthritis, at baseline (%)	1.2	0.6	3.5	3.5	2.2	*p* = 0.13
BMI 25 years change (kg/m^2^)	1.10	2.33	2.76	3.37	2.35	*p* < 0.001
HRT use (%)	26.3	32.0	35.5	41.5	23.8	*p* = 0.002
Number of chronic diseases (mean)	7.2	7.0	6.5	7.1	4.7	*p* = 0.41
Bone‐affecting diseases (%)	61.4	58.1	59.3	55.0	58.9	*p* = 0.28
Corticosteroid use (%)	19.3	15.1	15.1	15.8	19.5	*p* = 0.41

Significance between the quartiles: chi‐square analysis and ANOVA.

ANOVA = analysis of variance.

Prevalence of self‐reported HRT use was observed in 26.3% of the women in the lowest bone loss quartile (FQ1), 32% in FQ2, 35.5% in FQ3, and for 41.5% of women in FQ4 (*p* = 0.002 between FQ1 and FQ4). The duration of HRT during the 25‐year follow‐up was 65.8 months on average. The duration of HRT in the bone loss quartiles was FQ1 45.2, FQ2 54.8, FQ3 71.5, and FQ4 91.9 months, respectively. Therefore, the difference between FQ1 and FQ4 was 46.7 months. By contrast, the number of diseases did not differ between the quartiles (*p* = 0.41). No association was observed between corticosteroid use and bone loss rate (*p* = 0.41) in the prevalence of bone‐affecting diseases (*p* = 0.28), and menopausal status at baseline (*p* = 0.63) was not associated with the 25‐year bone loss rate. Furthermore, other risk factors investigated showed no significant association with bone loss rate (Table 2). In linear stepwise regression, BMI change (*p* < 0.001), BMI (*p* < 0.001), use of HRT (*p* = 0.010), and use of calcium/vitamin‐D (*p* = 0.029) showed statistical significance in prediction of BMD change.

## Discussion

The aim of this study was to study long‐term changes in postmenopausal BMD and to identify possible factors affecting bone loss. In this work we report the longest population‐based study of bone loss to date. The average bone loss rate of the total study population was found to be 10.1% ± 0.37% (mean ± SD) during the 25‐year follow‐up period, and 9.7% for those without any self‐reported medical condition. Previous studies have shown that bone loss decreases after perimenopause,^(^ ^5^, ^6^, ^8^ ^)^ and that over a 15‐year period, BMD at the femoral neck steadily decreases with age in postmenopausal women.^(^ ^9^ ^)^ We found that BMD at the femoral neck in postmenopausal women decreased during the 25‐year follow‐up with a steady pattern between ages 53.3 and 77.4 years. However, long‐term bone loss was much lower than in previous studies. The annual decline in BMD has previously been assumed to be 1.6% from the femoral neck and 3.1% from the lumbar spine in long‐term research.^(^ ^9^ ^)^ Our study showed that the BMD of the femoral neck decreases on average by 0.4% per year, which indicates that bone loss is 75% lower than previously suggested.^(^ ^9^ ^)^ The 25‐year BMD estimate was slightly higher in the 5‐year to 10‐year intervals (11.8–11.9% of the baseline BMD) than in the 15‐year to 25‐year intervals (8.9–10.1% of the baseline BMD). However, the observed bone loss in the total population was almost linear (beta = 0.07247, *t* = 0.507, *p* = 0.61).

An earlier study with a 15‐year follow‐up consisted of British women.^(^ ^27^, ^28^ ^)^ Inequalities in women's health between Britain and Finland have been reported, and one of the cultural differences at that time was that British women were more likely to remain as full‐time housewives or be only part‐time employees. Finnish women worked more full‐time. This may have some influence on bone loss differences, although on the other hand poor socioeconomic status appeared to be a risk factor for health in both societies. Furthermore, employed British women have reported less limiting chronic health disorders than Finnish full‐time workers.^(^ ^27^ ^)^

Factors influencing bone loss were investigated between the bone loss quartiles in our study. Higher baseline BMD was associated with a higher bone loss rate. However, women in the highest baseline BMD quartile did not reach the baseline BMD of the population mean during the 25‐year follow‐up. Lifestyle issues and genetics may both contribute to our finding that women with high baseline BMD have a higher bone loss rate. Regarding the factors influencing bone loss, the statistical analyses were based on either chi square or ANOVA. This includes the assumption that the predictors are independent.

Previous investigations have shown that HRT and increased BMI prevent bone loss.^(^ ^10^, ^11^, ^29^, ^30^, ^31^, ^32^ ^)^ Our study found similar results. The use of HRT appears to be associated with lower postmenopausal bone loss and the women in the lowest bone loss quartile had the greatest increase in BMI. Previous studies have revealed the association between increased BMI and lower bone loss.^(^ ^7^, ^31^, ^32^ ^)^ The number of diseases and the presence of bone‐affecting diseases did not have a significant effect on bone loss rate. Furthermore, the 9.7% bone loss in women without any medical condition is close to the mean bone loss of the total population (10.1%).

Women with high estrogen levels more probably have higher BMD. In the present study, the proportion of HRT users in the highest BMD quartile was 26.3%. The duration of HRT was 65.8 months on average. Between bone loss quartiles the duration of HRT was FQ1: 45.2, FQ2: 54.8, FQ3: 71.5; and FQ4: 91.9 months. Therefore the difference between FQ1 and FQ4 is 46.7 months. Both a lower baseline BMI and increase in BMI were associated with preventing bone loss. Previous trials^(^ ^33^ ^)^ have reported side effects of the long‐term HRT, which may have contributed to the higher bone loss rate after possible abrupt cessation of the therapy.

The characteristics of the participants in the 25‐year follow‐up differed from the characteristics of the baseline population. This may suggest that participants who continued throughout the 25‐year follow‐up may have been healthier in comparison to those who dropped out during the follow‐up. Hence, our results must be interpreted with some caution.

The most common reason for secondary osteoporosis is the use of corticosteroids.^(^ ^34^ ^)^ Corticosteroid‐induced bone loss is associated with the dose and duration of the treatment.^(^ ^34^, ^35^ ^)^ However, our study could not confirm that the use of corticosteroids was associated with increased bone loss. This may be due to issues related to the low dosage or short‐term use of corticosteroids.

The strengths of this study are a large base population with a long follow‐up duration. The study population represented a random population‐based cohort. The DXA measurement data at different time points was made comparable with the use of adequate correction functions. The possibility of bias due to informative dropout was explored with linear estimates. The attrition rate was found to be high, which is to be expected in such a long follow‐up study and taking into account the age group of the present study. As much as 35% of the dropouts of the measurement sample may be considered to be due to mortality or need for long‐term care.

At the beginning of the study, 62.1% of our study population was postmenopausal, and hence this follow‐up study predicts long‐term changes in BMD rather well, particularly in the case of postmenopausal women. The menopausal status at baseline was not associated with bone loss rate during the following 25 years, and the effect of menopausal transition on bone loss has been reported for this same cohort.^(^ ^7^ ^)^

The follow‐up study utilized four different generations of DXA scanners. The long‐term reproducibility error of the DXA scanner determined with phantom measurements was 0.4%. Despite in vivo cross‐calibration, a potential source of error from the scanner change cannot be excluded. In the present study, all scanners were matched to Lunar DPX‐IQ values with linear functions and mathematical models, as presented in previous studies.^(^ ^21^, ^22^, ^23^, ^24^ ^)^ However, a small inaccuracy may be present between the devices, although any potential error may be considered to be systematic.

Postmenopausal bone loss was found to be constant and appeared to be approximately 10% over 25 years. There is a lack of long‐term research describing postmenopausal bone loss over several decades, which warrants future research. The effect of long‐term bone loss on fracture patterns is beyond the scope of this study. Our findings suggest significantly lower bone loss in postmenopausal women than previously suggested.

## Disclosures

All the authors declare that they have no conflict of interest.

### Peer Review

The peer review history for this article is available at https://publons.com/publon/10.1002/jbmr.4444.

## Supporting information


**Supplemental Table S1.** Cumulative mortality and need for long‐term care of the OSTPRE population

## Data Availability

Data available on request from the authors
