# Supplementary material for: Characteristics of Long‐Term Femoral Neck Bone Loss in Postmenopausal Women: A 25‐Year Follow‐Up
Source: J Bone Miner Res. 2021 Oct 19;37(2):173–8. doi: 10.1002/jbmr.4444 (PMC9298425; doi:10.1002/jbmr.4444)
Supplement: Supplementary file 1 — Supplemental Table S1. Cumulative mortality and need for long‐term care of the OSTPRE population [file JBMR-37-173-s001.docx]

Supplemental table 1: Cumulative mortality and need for long-term care of the OSTPRE population

| **Years since 1989** | **Cum Deaths (%)** | **Cum long-term care (%)** | **Both (%)** |
| --- | --- | --- | --- |
| 1 | 0.4 | 0.8 | 1.2 |
| 2 | 0.7 | 0.9 | 1.6 |
| 3 | 1.2 | 1.0 | 2.1 |
| 4 | 1.5 | 1.1 | 2.6 |
| 5 | 1.8 | 1.3 | 3.1 |
| 6 | 2.2 | 1.4 | 3.6 |
| 7 | 2.7 | 1.6 | 4.4 |
| 8 | 3.1 | 1.8 | 4.9 |
| 9 | 3.5 | 2.0 | 5.5 |
| 10 | 4.0 | 2.1 | 6.1 |
| 11 | 4.5 | 2.4 | 6.9 |
| 12 | 5.1 | 2.6 | 7.7 |
| 13 | 5.7 | 2.9 | 8.6 |
| 14 | 6.3 | 3.2 | 9.5 |
| 15 | 7.0 | 3.4 | 10.5 |
| 16 | 7.8 | 3.7 | 11.5 |
| 17 | 8.4 | 4.0 | 12.5 |
| 18 | 9.2 | 4.5 | 13.7 |
| 19 | 10.0 | 5.1 | 15.1 |
| 20 | 10.9 | 5.8 | 16.7 |
| 21 | 11.9 | 6.5 | 18.5 |
| 22 | 13.0 | 7.4 | 20.4 |
| 23 | 14.0 | 8.1 | 22.1 |
| 24 | 15.2 | 8.7 | 24.0 |
| 25 | 16.6 | 9.5 | 26.1 |
